# Pornography addiction: A neuroscience perspective

**DOI:** 10.4103/2152-7806.76977

**Published:** 2011-02-21

**Authors:** Donald L. Hilton, Clark Watts

**Affiliations:** Department of Neurosurgery, University of Texas Health Science Center at San Antonio, San Antonio, TX, USA; 1 Department of Neurosurgery, University of Texas School of Law, Austin, TX, USA

A significant postulate of this commentary is that all addictions create, in addition to chemical changes in the brain, anatomical and pathological changes which result in various manifestations of cerebral dysfunction collectively labeled hypofrontal syndromes. In these syndromes, the underlying defect, reduced to its simplest description, is damage to the “braking system” of the brain. They are well known to clinical neuroscientists, especially neurologists and neurosurgeons, for they are also seen with tumors, strokes, and trauma. Indeed, anatomically, loss of these frontal control systems is most apparent following trauma, exemplified by progressive atrophy of the frontal lobes seen in serial MRI scans over time.

Although the key elements of hypofrontal syndromes—impulsivity, compulsivity, emotional lability, impaired judgment—are well described, much of the process is still unknown. One emerging aspect of these hypofrontal states is their similarity to findings in addictive patients. Addressing hypofrontality, Fowler *et al*. noted, “studies of addicts show reduced cellular activity in the orbitofrontal cortex, a brain area…[relied upon]…to make strategic, rather than impulsive, decisions. *Patients with traumatic injuries to this area of the brain display problems–aggressiveness, poor judgment of future consequences, inability to inhibit inappropriate responses that are similar to those observed in substance abusers.*”[8] (emphasis added).

In 2002, a study on cocaine addiction demonstrated measurable volume loss in several areas of the brain, including the frontal lobes.[9] The study technique was an MRI-based protocol, voxel-based morphometry (VBM), where 1 mm cubes of brain are quantified and compared. Another VBM study was published in 2004 on methamphetamine, with very similar findings.[27] While interesting, these findings may not be surprising to either the scientist or the layperson, as these are “real drugs” used illicitly. Nevertheless, it was noteworthy that addiction could produce measurable, anatomical change in the brain.

Even more instructive are similar findings seen with the abuse of a normal biological behavior, eating, leading to addiction and obesity. In 2006, a VBM study was published looking specifically at obesity, and the results were very similar to the cocaine and methamphetamine studies.[20] The obesity study demonstrated multiple areas of volume loss, particularly in the frontal lobes, areas associated with judgment and control. This study is significant in demonstrating visible damage in a natural endogenous addiction, as opposed to an exogenous drug addiction. Furthermore, it is easy to accept intuitively because the effects of overeating can be seen in the obese person.

Eating, of course, is essential to individual survival, necessary for survival of the species. Another activity necessary for survival of the species is sex, an observation which leads to a series of logical questions derived from the work on obesity. Would the findings seen in eating addiction be seen in excessive sexual behavior? Can sex be addictive in the neurological sense? If so, are there associated with the addiction anatomical changes in the brain seen with other addictions?A recent study supports growing evidence that compulsive sexuality can indeed be addictive. In 2007, a VBM study out of Germany looked specifically at pedophilia, and demonstrated almost identical finding to the cocaine, methamphetamine, and obesity studies.[25] It concludes for the first time that a sexual compulsion can cause physical, anatomic change in the brain, the hallmark of brain addiction. A preliminary study showed frontal dysfunction specifically in patients unable to control their sexual behavior.[16] This study used diffusion MRI to evaluate function of nerve transmission through white matter. It demonstrated abnormality in the superior frontal region, an area associated with compulsivity.

A decade ago Dr. Howard Shaffer at Harvard wrote, “I had great difficulty with my own colleagues when I suggested that a lot of addiction is the result of experience … repetitive, high-emotion, high-frequency experience. But it’s become clear that neuroadaptation—that is, changes in neural circuitry that help perpetuate the behavior—occurs even in the absence of drug-taking.”[13] More recently he wrote, “Although it is possible to debate whether we should include substance or process addictions within the kingdom of addiction, technically there is little choice. Just as the use of exogenous substances precipitate impostor molecules vying for receptor sites within the brain, human activities stimulate naturally occurring neurotransmitters. The activity of these naturally occurring psychoactive substances likely will be determined as important mediators of many process addictions.”[24]

In 2005, Dr. Eric Nestler wrote a landmark paper describing all addiction as a dysfunction of the mesolimbic reward centers of the brain. Addiction occurs when pleasure/reward pathways are hijacked by exogenous drugs such as cocaine or opioids, or by natural processes essential and inherent to survival such as food and sex. The same dopaminergic systems include the ventral tegmental area with its projections to the nucleus accumbens and other striatal salience centers. He wrote, “Growing evidence indicates that the VTA-NAc pathway and the other limbic regions cited above similarly mediate, at least in part, the acute positive emotional effects of natural rewards, such as food, sex and social interactions. These same regions have also been implicated in the so-called ‘natural addictions’ (that is, compulsive consumption for natural rewards) such as pathological overeating, pathological gambling, and sexual addictions. Preliminary findings suggest that shared pathways may be involved: (an example is) cross-sensitization that occurs between natural rewards and drugs of abuse.”[18]

This attention to process (or natural) addictions requires focus on metabolic dysfunction in the mesolimbic salience pathways. Just as exogenously administered drugs cause downgrading of dopamine receptors in the nucleus accumbens in addiction, evidence supports endogenously functioning neurotransmitters causing similar pathology.

The prestigious Royal Society of London, founded in the 1660s, publishes the longest running scientific journal in the world. In a recent issue of the *Philosophical Transactions of the Royal Society*, the current state of the understanding of addiction was reported as it was discussed by some of the world’s leading addiction scientists at a meeting of the Society. The title of the journal issue reporting the meeting was “The neurobiology of addiction—new vistas.” Interestingly, of the 17 articles, two were specifically concerned with evidence for natural addiction: pathologic gambling[23] and overeating.[28] A third paper, addressing animal models of drug and natural addiction, related to DeltaFosB.[19] DeltaFosB is a protein studied by Nestler that appears to be over-expressed in the neurons of addicted subjects. It was first found in the neurons of animals studied in drug addiction[17] but has now been found in the nucleus accumbens related to over-consumption of natural rewards.[18] A recent paper investigating DeltaFosB and its role in over-consumption of two natural rewards, eating, and sexuality, concludes: …the work presented here provides evidence that, in addition to drugs of abuse, natural rewards induce ∆FosB levels in the Nac…our results raise the possibility that ∆FosB induction in the NAc may mediate not only key aspects of drug addiction, but also aspects of so-called natural addictions involving compulsive consumption of natural rewards.[29]

Even more pertinent are recent papers published in 2010 describing the effect of sexuality on neuroplasticity. In one study, sexual experience has been shown to induce alterations in medium spiny neurons in the nucleus accumbens similar to those seen with drugs of abuse.[21] Another study found that sexuality specifically increases DeltaFosB in the nucleus accumbens, and serves a role as a mediator in natural reward memory. This study also found that overexpression of DeltaFosB induced a hypersexual syndrome.[22] As Dr. Nestler said, DeltaFosB may thus become a “biomarker to assess the state of activation of an individual’s reward circuitry, as well as the degree to which an individual is ‘addicted’, both during the development of an addiction and its gradual waning during extended withdrawal or treatment.”[22]

Dr. Nora Volkow, Head of the National Institute on Drug Abuse (NIDA), and one of the most published and respected scientists in the field of addiction is, in recognition of the change in the understanding of natural addiction, advocating changing the name of the NIDA to the National Institute on Diseases of Addiction, as quoted in the journal *Science*: “NIDA Director Nora Volkow also felt that her institute’s name should encompass *addictions such as pornography*, gambling, and food, says NIDA adviser Glen Hanson. ‘She would like to send the message that [we should] look at the whole field.’”[7] (emphasis added).

With the increasing evidence that overeating can be an actual addiction as defined by measurable, verifiable changes in the limbic salience centers, our attention to this problem is appropriately increasing. Yet sexuality, with its moral ties, is handled much less objectively in scientific debate. This was apparent in the aftermath of the Hogg study published in 1997, which demonstrated a 20-year decrease in life expectancy for male homosexuals.[12] The authors, apparently feeling social pressure, issued a clarification to avoid being labeled what they called “homophobic.”[11] That a science journal would publish such an apology of sorts is also noteworthy. We believe, however, with the preceding foundation it is time to begin serious discussions of sexual addiction and its components such as pornography.

The proposed DSM-5, slated to publish in May of 2014, contains in this new addition the diagnosis of Hypersexual Disorder, which includes problematic, compulsive pornography use.[1] Bostwick and Bucci, in their report out of the Mayo Clinic on treating Internet pornography addiction with naltrexone, wrote “…cellular adaptations in the (pornography) addict’s PFC result in increased salience of drug-associated stimuli, decreased salience of non-drug stimuli, and decreased interest in pursuing goal-directed activities central to survival.”[3]

In 2006 world pornography revenue was 97 billion dollars, more than Microsoft, Google, Amazon, eBay, Yahoo, Apple, and Netflix combined.[14] This is no casual, inconsequential phenomenon, yet there is a tendency to trivialize the possible social and biologic effects of pornography. The sex industry has successfully characterized any objection to pornography as being from the religious/moral perspective; they then dismiss these objections as First Amendment infringements. If pornography addiction is viewed objectively, evidence indicates that it does indeed cause harm in humans with regard to pair-bonding.[2] The correlation (85%) between viewing child pornography and participating in actual sexual relations with children was demonstrated by Bourke and Hernandez.[4] The difficulty in objective peer-reviewed discussion of this topic is again illustrated by the attempted suppression of this data on social grounds.[15] The recent meta-analysis by Hald *et al*. strongly supports and clarifies previous data demonstrating correlation with regard to pornography inducing violence attitudes against women.[10] With such strong correlative data, it is irresponsible not to address the likely possibility of causation in these regards. Reviewing this data in the context of current usage patterns is particularly concerning; 87% of college age men view pornography, 50% weekly and 20 daily or every other day, with 31% of women viewing as well.[5] The predictive effect of pornography on sexual behavior in adolescents has also been demonstrated.[6]

Certainly our role as healers suggests we can do more to investigate and treat human pathology related to this new entity of process or natural addiction, particularly given the growing weight of evidence supporting the neural basis of all addictive processes. Just as we consider food addiction as having a biologic basis, with no moral overlay or value-laden terminology, it is time we looked at pornography and other forms of sexual addiction with the same objective eye. Currently, social pressures relegate the management of pornography primarily to proceedings in civil or in criminal judicial venues.[26] This commentary is not a plea to change those practices any time soon. It is a statement that seeks to encourage an examination by medicine in general and the clinical neuroscience specialties specifically of the role for medical treatment in the management of the addictive nature of the pathology of pornography.

In concluding this thought, a Public Health profile of pornography might be useful. Any such profile by its nature will be somewhat primitive because of the current status of the knowledge of the addiction and the environment in which it occurs. Table 1 is an attempt to provide such a profile of the case of pornography, using as a model the investigation of an outbreak of cholera in London in 1854, when the understanding of the Public Health implications of cholera by medicine was perhaps as primitive as that of pornography today. While noting the huge contribution by the industry of the physical material of pornography that will need to be addressed through nonmedical resources, it also suggests a place for medicine in management of the addiction.

**Table 1:** Pornography a public health profile

Characteristics	Model*	Case**

Place/date	London (1854)	USA (2010)
Critical defect	Contaminated water supply	Public attitude: constrained indulgence
Fascilitator	Water supply	Purveyors
Agent	V. cholerae	Raw material
Clinical condition	Cholera	Drug imbalance (brain)
Disease category	Infection	Addiction
Outcome with-out treatment	Biological deterioration and death	Hypofrontal syndromes (?)
At Risk	Close associates (and family)	Poorly understood
Treatment	General medical principles	Awaiting consensus

**Extra-medical actions**		

Public health challenge	Prevent contamination	Appropriate education (social, political, biological)
Agency	Political (governmental)	Venue-appropriate institutions
		

* S. Johnson. The Ghost Map. Riverhead Books. New York. 2006

** extrapolated from various popular and scientific commentaries, some referenced in this paper

Table 1 is submitted to help launch the debate of these questions: what are the medical implications of pornography given current evidence supporting an addictive model?, how should they be addressed relative to the major nonmedical resources required for a comprehensive societal response to this problem?, how may the experience of clinical neuroscientists with hypofrontal syndromes be brought collaboratively in support of those scientists experienced in addiction?
